# Underweight and associated factors among lactating women in Uganda: Evidence from the Uganda demographic health survey 2016

**DOI:** 10.1002/hsr2.356

**Published:** 2021-08-13

**Authors:** Quraish Sserwanja, Joseph Kawuki, Linet M. Mutisya, Milton W. Musaba, Mathew Kagwisagye, Ivan Arinda Kato, David Mukunya

**Affiliations:** ^1^ Programmes Department GOAL Khartoum Sudan; ^2^ Centre for Health Behaviours Research, Jockey Club School of Public Health and Primary Care The Chinese University of Hong Kong Hong Kong‐SAR China; ^3^ Mama and Family Project Swedish Organization for Global Health Mayuge Uganda; ^4^ Department of Obstetrics and Gynaecology Mbale Regional Referral and Teaching Hospital Mbale Uganda; ^5^ Department of Obstetrics and Gynaecology Busitema University Tororo Uganda; ^6^ Department of Epidemiology and Biostatistics Makerere University School of Public Health Kampala Uganda; ^7^ Department of Research Sanyu Africa Research Institute Mbale Uganda; ^8^ Department of Community and Public Health Busitema University Tororo Uganda

**Keywords:** lactating‐women, prevalence, Uganda, underweight

## Abstract

**Background:**

Lactating mothers are at increased risk of being underweight because of the physiological changes that lead to disproportionately higher energy and nutrient requirements compared to their non‐pregnant and non‐lactating counterparts.

**Objective:**

We aimed to determine the prevalence and factors associated with being underweight among lactating women in Uganda.

**Methods:**

We used the Uganda Demographic and Health Survey (UDHS) 2016 data of 1356 women aged 20 to 49 years. Multistage stratified sampling was used to select study participants. The data were collected using validated questionnaires. We used multivariable logistic regression to determine factors associated with underweight among 20 to 49‐year‐old lactating women in Uganda.

**Results:**

The prevalence of underweight was 8.2% (111/1356) (95% confidence interval, [CI]: 7.0‐10.0). Women who had no education were 10.21 (adjusted odds ratio, [AOR] = 10.21; 95% CI: 1.61‐64.74) times as likely to be underweight as those who had higher (post‐secondary) education levels. Women who were not working were 50% (AOR = 0.50; 95% CI: 0.26‐0.94) less likely to be underweight compared to those who were working. Women in the Western (AOR = 0.15; 95% CI: 0.07‐0.32), Eastern (AOR = 0.34; 95% CI: 0.18‐0.66), and Central (AOR = 0.30; 95% CI: 0.12‐0.74) regions were 85%, 66% and 70% respectively less likely to be underweight compared to those in the Northern region.

**Conclusion:**

Based on the findings of this and other studies, it is important for the different stakeholders to design targeted nutrition programs for lactating women particularly those with low levels of education and those from the Northern region.

## 1 INTRODUCTION

Maternal and child nutrition are good indicators of a society's overall wellbeing.^1^ Globally, about 10% of women aged 20 to 49 years are underweight^2^ with the greatest burden observed in low‐income countries.^3^ Underweight is considered an indicator of undernutrition in an adult with no underlying comorbidities and is defined as body mass index (BMI) below 18.5 kg/m^2^.^4^, ^5^ Pregnancy and lactation are nutritionally demanding periods due to increased calorie and nutrient requirements. This can be detrimental to a woman's health if food intake is not commensurate.^1^, ^6^

The World Health Organization (WHO) recommends up to 2 years of breastfeeding, with exclusive breastfeeding for the first 6 months of a baby's life.^7^ The production of breast milk largely depends on the nutritional status of the mother, hence the need for adequate nutrition during lactation.^7^ In addition, this period is enough to ensure full recovery from a preceding pregnancy and reversal of the associated physiological changes, the WHO recommends a birth‐spacing interval of at least 24 months.^8^ Maternal underweight negatively affects the quality and quantity of nutrients in breast milk leading to increased risk of child morbidity, mortality, and adverse long‐term effects on the child's health.^9^, ^10^ Under‐nutrition among lactating mothers induces nutrition‐related metabolic disturbances in early infancy and irreversible physiologic alterations in infants.^11^

In low‐income countries, women are at a high risk of unmet nutrient requirements because of inadequate food supply mainly attributed to financial constraints.^12^ Most women in Uganda are economically disadvantaged; they have less control of resources such as land, are much more involved in unpaid care work, their work hours are longer compared to men, and they have less access to credit services.^13^ Majority of previous studies in Uganda have focused on prenatal and under‐five nutrition and overlooked lactating women despite studies in other countries showing a high prevalence of underweight among lactating women.^10^, ^14^ This knowledge gap impedes the designing of national nutrition policies for pregnant and lactating women. This study aimed to determine the prevalence and factors associated with undernutrition among lactating women in Uganda.

## 2 MATERIAL AND METHODS

### 2.1 Study data

We used secondary data from the 2016 Uganda demographic health survey (UDHS) collected between June 20 to December 16, 2016.^15^ UDHS is a periodical national survey, conducted every 5 years by the Uganda Bureau of Statistics as part of the international MEASURE Demographic Health Surveys (DHS) with the support of ICF International and United States Agency for International Development (USAID).^15^ The survey had four different questionnaires: (a) household questionnaire (household and environment data), (b) women's questionnaire (women's characteristics including reproductive health, domestic violence, and nutrition), (c) men's questionnaire (men's health indicators' data), and (d) biomarker questionnaire (anthropometry and biochemical tests).^15^ Weight was recorded in kilograms, rounded off to the nearest single decimal point, and was measured using an electronic scale (SECA 878) while height was recorded in centimeters and similarly rounded off.^15^

#### 2.1.1 Study setting

As of July 2018, Uganda (241 551 km^2^) had a population of 40 853 749 million people with approximately 23.8% residing in urban areas.^16^, ^17^ Uganda's health system has six levels ranging from the highest level of national referral hospitals to the lowest level at the community level.^18^ Agriculture contributes about 24% of the country's gross domestic product (GDP), providing half of the export earnings, and is the primary source of income for the majority of Ugandans.^17^

### 2.2 Study sampling and participants

Samples were collected using a stratified two‐stage cluster sampling design with census enumeration areas as the primary sampling units.^15^ The first stage of sampling involved selecting 697 enumeration areas (EAs), including 162 urban and 535 rural enumeration areas selected from the list of the 2014 population and housing census sample frame.^15^ One enumeration area in the Acholi region was excluded due to land disputes hence ending up with 696 EAs. Enumeration areas with over 300 households were segmented and only one segment was selected with probability proportional to the segment size as this helped minimize the burden of the household listing.^15^ The enumeration areas that were involved in the survey were chosen independently from each stratum with probability proportional to size. The second stage of sampling involved the selection of households through equal probability systematic sampling. A list containing all households and maps in the selected enumeration area was made available, and households that were in institutional living arrangements were excluded.^15^ Women aged 15 to 49 years who were either permanent residents or slept in the selected household the night before were eligible for inclusion in Uganda's demographic health survey 2016.^15^ During the survey, anthropometric measurements were taken by trained technicians in about a third of the sampled women.^15^ Our secondary analysis only considered 20 to 49‐year‐old lactating women and excluded 15 to 19‐year‐old women (adolescents) because the recommended anthropometric indicators for assessing underweight for those above 20 years (BMI and Height) are different from those of adolescents (BMI for age and Height for age) and cannot be directly compared. Of the 18 506 women who consented and filled in the questionnaires, 14 242 were aged 20 to 49 years and of these, 4731 were selected for anthropometric assessment of which 4640 had their measures taken. From this group, 1356 were lactating women (Table 1), and 289 of them were excluded from logistic regression analysis because of being overweight or obese, leaving a final weighted sample of 1067 women. The sample selection process is summarized by the consort flow diagram in Data S1. To maintain the representativeness of the sample and account for possible differences in response rates across regions, sampling weights were used.

**TABLE 1:** Sociodemographic characteristics of lactating Ugandan women aged 20 to 49 years as per the 2016 UDHS

Characteristics	N = 1356	%
Age		
20 to 29	834	61.5
30 to 39	441	32.5
40 to 49	81	06.0
Residence		
Rural	1092	80.5
Urban	264	19.5
Region		
Western	361	26.6
Eastern	272	20.0
Central	308	22.7
Northern	415	30.6
Sex household head		
Female	343	25.3
Male	1013	74.7
Household size		
6 and Above	738	54.5
Less than 6	618	45.5
Working status^a^		
Not working	233	17.2
Working	1122	82.7
Marital status		
Married	1194	88.1
Not married	161	11.9
Education level		
No education	166	12.2
Primary education	807	59.5
Secondary education	280	20.7
Higher	103	07.7
Wealth index		
Poorest	330	24.3
Poorer	291	21.4
Middle	268	19.7
Richer	227	16.8
Richest	241	17.7
Underweight		
Yes	111	08.2
No	1245	91.8
ANC frequency^b^		
Less than 4 visits	547	40.4
4 visits and above	806	59.6

^a^ Working status had one missing value.

^b^ ANC frequency had two missing values.

### 2.3 Outcome variables

In this study, underweight was the primary outcome variable of interest, defined as body mass index (BMI) less than 18.50 kg/m^2^.^4^ Other BMI categories were defined as follows; normal = between 18.50 and 24.99 kg/m^2^, overweight = between 25.0 and 29.99 kg/m^2^ and obesity = above 29.99 kg/m^2^.^5^ In the logistic regression analysis, we only considered underweight women and those with normal BMI and excluded those who were overweight and obese.

### 2.4 Explanatory variables

This study included determinants of underweight based on evidence from the available literature. These factors were divided into individual (age, marital status, working status, frequency of antenatal care [ANC] utilization and education level), household (wealth index, household size and sex of household head), and community (region and residence) levels. The “Wealth index” is a measure of relative household economic status and was calculated by DHS from information on household asset ownership using Principal Component Analysis.^15^, ^19^ Different household assets were used to calculate separate wealth indices for rural and urban areas which were combined to form a national wealth index and then divided into quintiles namely; the poorest, the poorer, the middle, the richer, and the richest.^15^, ^19^ Place of Residence was aggregated as urban or rural. The national region was categorized as Northern, Central, Eastern, and Western. Level of Education was categorized into; no education, primary education, secondary, and higher education. Age was categorized into three groups; 20 to 29, 30 to 39, and 40 to 49 years. Household Size was categorized as less than six members and six and above members based on the national and dataset average of six members per household. The sex of Household Head was categorized as male or female. Working status was categorized as: not working and working. Marital Status was categorized into married, and this included those in formal and informal unions, and not married. ANC utilization frequency was divided into less than four visits and four and above visits.

### 2.5 Statistical analysis

We used the SPSS analytic software version 25.0 complex samples package for this analysis. The use of this complex samples package accounted for the complex survey sampling while the use of sample weighted data accounted for the unequal probability sampling in different strata. Frequency tables and proportions were used to describe categorical variables while means and standard deviations for continuous variables. Each exposure was assessed separately for its association with the outcome using bivariable logistic regression, and we present crude odds ratio (COR) and their 95% confidence interval (CI). Variables included in our multivariable model were determined a priori during the literature review.^20^ Factors included in our model were: age, level of education, residence, region, household size and sex of household head, working and marital status, wealth index, and frequency of ANC utilization.^10^, ^14^, ^21^ We used variance inflation factor to rule out multi‐co‐linearity and Hosmer and Lemeshow goodness of fit to test the adequacy of the model.^12^ Adjusted odds ratios (AOR), 95% Confidence Intervals (CI) and *P*‐values are presented.

### 2.6 Ethical considerations

High international ethical standards are ensured for MEASURE DHS surveys as ethical approval from the country is obtained from a national ethical review board and local authorities before implementing the survey and well‐informed verbal consent is sought from the respondents prior to data collection.^15^, ^19^ This data set was obtained from the MEASURE DHS website (URL: https://www.dhsprogram.com/data/available-datasets.cfm) after getting official permission to utilize it. As such, there was no need for ethical approval to conduct a secondary analysis of publicly available data.

## 3 RESULTS

### 3.1 Sociodemographic characteristics of study participants

A total of 1356 lactating women were included in this study and over half of them were aged between 20 and 29 years. A great majority resided in rural areas (80.5%), were currently working (82.7%) and married (88.1%). Slightly more than half of the women lived in households with more than six members (54.5%) and had primary education as the highest level (59.5%) and most resided in male‐headed households (74.7%). The Northern region had the highest proportion of respondents (30.6%) while Eastern had the lowest (20.0%). Study participants are almost equally distributed in each wealth quintile. More details about participants' characteristics are contained in Table 1.

### 3.2 Nutritional status of women

The mean age, weight, height, household size, and BMI were 28.44 ± 6.21, 57.83 ± 10.66, 159.09 ± 6.39, 6.23 ± 2.72, and 22.83 ± 3.89 respectively. The prevalence of underweight was 8.2% (95% CI: 7.0‐10.0), and of these, 75.9% were mildly underweight, 20.5% moderately underweight, and 3.6% severely underweight. The prevalence of normal BMI was 70.5% (95% CI: 68.1‐72.9) while overweight was 15.8% (95% CI: 13.7‐17.5) and obesity was 5.6% (95% CI: 4.4‐6.9). Table 2 shows the distribution of underweight by sociodemographic characteristics.

**TABLE 2:** Distribution of underweight by sociodemographic characteristics among lactating Ugandan women aged 20 to 49 years

Characteristics	Underweight n = 111	Not underweight n = 956	*P*‐value
Household head			.475
Female	30 (26.7)	229 (24.0)	
Male	81 (73.3)	727 (76.0)	
Wealth index			.001
Poorest	50 (45.0)	258 (27.0)	
Poorer	26 (23.7)	228 (23.8)	
Middle	15 (13.3)	194 (20.3)	
Richer	15 (13.3)	156 (16.3)	
Richest	5 (04.7)	120 (12.6)	
Working status^a^			.019^b^
Not working	9 (8.1)	159 (16.6)	
Working	102 (91.9)	797 (83.4)	
Education level			.001^b^
No education	24 (21.8)	118 (12.2)	
Primary	72 (65.5)	584 (61.1)	
Secondary	13 (11.8)	193 (20.2)	
Higher	1 (0.9)	62 (6.5)	
Region			<.001^b^
Western	9 (8.0)	261 (27.3)	
Eastern	16 (14.7)	217 (22.7)	
Central	11 (9.8)	176 (18.4)	
Northern	75 (67.6)	302 (31.6)	
Marital status			.422
Married	100 (90.1)	836 (87.4)	
Not married	11 (9.9)	120 (12.6)	
Age			.737
20 to 29	68 (61.3)	621 (65.0)	
30 to 39	37 (33.3)	286 (29.9)	
40 to 49	6 (5.4)	49 (5.1)	
Residence			.366
Rural	97 (87.5)	804 (84.1)	
Urban	14 (12.5)	152 (15.9)	
Household size			.745
Six and Above	58 (51.8)	511 (53.5)	
Less than 6	53 (48.2)	445 (46.5)	
ANC frequency			.151^b^
Less than 4 visits	98 (88.3)	793 (82.9)	
4 visits and above	13 (11.7)	163 (17.1)	

^a^ Working status had one missing value.

^b^ Significant at *P*‐value <.20.

### 3.3 Factors associated with underweight

In the final logistic regression model, factors associated with underweight were region, education level, and working status as shown in Table 3. Women who had no education were 10.21 times more likely to be underweight compared to those who had higher (post‐secondary) education level. Women who were not working were 50% less likely to be underweight compared to those who were working. Women in the Western, Eastern, and Central regions were 85%, 66%, and 70% less likely respectively to be underweight compared to those in the Northern region.

**TABLE 3:** Associated factors of underweight among lactating Ugandan women aged 20 to 49 years

Characteristics	Crude (n = 1067) OR (95%CI)	*P*‐value	Adjusted (n = 1067) AOR (95%CI)	*P*‐value
Education level		**.002**		**.030**
Higher	1		1	
Secondary	3.53 (0.72‐17.18)		3.98 (0.63‐25.20)	
Primary	**6.32 (1.42‐28.06)**		**6.35 (1.13‐35.76)**	
No education	**10.67 (2.29‐49.74)**		**10.21 (1.61‐64.74)**	
Working status		**.010**		**.032**
Working	1		1	
Not working	**0.44 (0.24‐0.82)**		**0.50 (0.26–0.94)**	
Region		**<.001**		**<.001**
Northern	1		1	
Western	**0.14 (0.06‐0.29)**		**0.15 (0.07–0.32)**	
Eastern	**0.30 (0.16‐0.56)**		**0.34 (0.18‐0.66)**	
Central	**0.25 (0.10‐0.39)**		**0.30 (0.12‐0.74)**	
Wealth index		**.005**		.939
Richest	1		1	
Richer	2.18 (0.60‐7.87)		1.21 (0.27‐5.39)	
Middle	1.72 (0.48‐6.22)		0.82 (0.18‐3.69)	
Poorer	2.64 (0.78‐8.99)		0.98 (0.22‐4.39)	
Poorest	**4.42 (1.34‐14.61)**		0.98 (0.22‐4.26)	
ANC frequency		.136		.341
4 and above	1		1	
Less than 4	1.62 (0.86‐3.05)		1.36 (0.72‐2.59)	
Residence		.420		.669
Urban	**1**		1	
Rural	1.32 (0.67‐2.61)		0.84 (0.37‐1.89)	
Sex of household head		.533		.902
Male	1		1	
Female	1.15 (0.74‐1.80)		1.03 (0.64‐1.67)	
Household size		.746		.199
Less than 6	1		1	
Six and above	0.94 (0.63‐1.39)		0.74 (0.47‐1.17)	
Marital status		.432		.437
Not married	1		1	
Married	1.30 (0.67‐2.52)		1.33 (0.65‐2.71)	
Age		.773		.997
40 to 49	1		1	
30 to 39	1.02 (0.43‐2.42)		1.03 (0.42‐2.52)	
20 to 29	0.88 (0.39‐1.98)		1.01 (0.40‐2.58)	

*Note*: bold = Significant at *P*‐value <.05, Final model ‐ Adjusted for ANC frequency, region, working status, education level and wealth index.

Abbreviations: AOR, Adjusted odds ratio; COR, Crude Odds Ratio.

## 4 DISCUSSION

This study investigated the prevalence and factors associated with underweight among lactating Ugandan women aged between 20 and 49 years. Based on the anthropometric assessment, 8.2% (95% CI: 7.0‐10.0) of the lactating women were underweight, a prevalence that is within the standard acceptable malnutrition rate of less than 10%.^22^ Of the 8.2%, 3.6% were severely underweight, 20.5% were moderately underweight and 75.9% were mildly underweight. This prevalence is lower than studies conducted among lactating women in Ethiopia^6^, ^9^, ^12^ and Vietnam^14^ but higher than a study done in Nigeria.^21^ The difference in the prevalence of underweight between the current study and the other studies above could be attributed to the differences in the sociodemographic and economic characteristics between these study areas.

Underweight was significantly associated with working status, education level, and region. Women who belonged to the western, eastern, and central regions of the country were less likely to be underweight compared to those in the Northern region. Region of residence has also been shown to be associated with undernutrition in similar low‐income African settings.^4^, ^23^ Some parts of Northern Uganda especially the North‐Eastern area are among the most food‐insecure with limited resources.^24^, ^25^ This could be attributed to the fact that this region experienced a long civil war which greatly affected their agricultural production and the economy compared to the other regions of the country that have been stable without civil conflicts.^26^ The decreased agricultural production and poor economy of this region induced food insecurity by reducing local food production, hence decreased availability and access to food.^27^ This leads to inadequate food, in both quality and quantity, risking them to be underweight. In addition, most people in the Northern region, unlike the other regions are pastoral communities (some are nomadic) and this may negatively affect their consumption of food crops because they mostly focus on pastoral activities. Pastoralism has been shown to increase the risk of being underweight among Ethiopian pastoral communities.^4^

Women who were not working were 49% less likely to be underweight compared to those who were working. Working status has been found to be associated with being underweight in Ethiopia, Senegal, and Cambodia.^12^, ^28^, ^29^ The workload due to labor‐intensive activities increase energy expenditure which further predisposes these women to underweight. Similarly, a study done among lactating Senegalese women showed weight loss due to negative energy balance associated with agricultural labor,^28^ which agricultural activities are the commonest source of income in Uganda.^17^ Another possible reason could be tight working schedules that affect the dietary patterns of lactating mothers, which coupled with the increased nutrition needs, makes lactating mothers prone to underweight.^30^, ^31^

Similar to studies done in Ethiopia,^12^ Nepal,^2^ Tanzania,^4^ and Iran,^1^ education level in this study was significantly associated with the nutritional status. Women who had no education and those with only primary level were 9.27 and 6 times more likely respectively, to be underweight compared to those that had a post‐secondary level of education. Education level has been shown to be one of the indicators of women empowerment hence less educated women tend to have less knowledge on nutrition, be less empowered to access economic resources, and also less likely to make the right health decisions.^2^ This negatively affects the use of health‐care facilities, accessibility of nutritious food, better health‐promoting behaviors, and management of the limited household resources. All the aforementioned predispose women to inadequate dietary intake due to poor dietary consumption patterns like skipping meals and /or having unbalanced diets and eventually leading to underweight.

### 4.1 Strengths

Standardized procedures are a requirement of DHS surveys in data collection and validated questionnaires were used which ensured the internal and external validity of the results.

Secondly, we used a nationally representative sample and weighed the data for analysis and therefore our results are generalized to all Ugandan women aged 20 to 49 years.

### 4.2 Limitations

The cross‐sectional design is limited by lack of temporality hence causal inferences cannot be made. Most data on the predictors were based on self‐reporting and could not be verified through records, which could have led to socially acceptable answers hence information bias. Other important predictors of underweight such as dietary diversity score, nutritional knowledge, co‐morbidities, and food security were not collected.

## 5 CONCLUSION

Our study established that the factors associated with being underweight among Ugandan lactating women were level of education, working status, and region. Based on the findings of this and other studies, it is important for the different stakeholders to design targeted nutrition programs for lactating women with special emphasis on working, women from the Northern region, and those with low levels of education.

## FUNDING

No funding was obtained for this study.

## CONFLICT OF INTEREST

The authors declared no potential conflicts of interest with respect to the research, authorship, and/or publication of this article.

## TRANSPARENCY STATEMENT

The manuscript is an honest, accurate, and transparent account of the study being reported; and no important aspects of the study have been omitted.

## AUTHORS' CONTRIBUTIONS

Conceptualization: Quraish Sserwanja, David Mukunya.

Formal Analysis: Quraish Sserwanja.

Methodology: Quraish Sserwanja, Joseph Kawuki, Linet M. Mutisya, Milton W. Musaba, Mathew Kagwisagye, Ivan Arinda Kato, David Mukunya.

Writing ‐ Original Draft Preparation: Quraish Sserwanja.

Writing ‐ Review & Editing: Quraish Sserwanja, Joseph Kawuki, Linet M. Mutisya, Milton W. Musaba, Mathew Kagwisagye, Ivan Arinda Kato, David Mukunya.

All authors have read and approved the final manuscript version of the manuscript.

The corresponding author had full access to all of the data in this study and takes complete responsibility for the integrity of the data and the accuracy of the data analysis.

## Supporting information

**Data S1.** Supporting Information.

## Data Availability

Access to the DHS data sets is openly available upon requests made to MEASURE DHS on their website (URL: https://www.dhsprogram.com/data/available-datasets.cfm).
