# Supplementary material for: Underweight and associated factors among lactating women in Uganda: Evidence from the Uganda demographic health survey 2016
Source: Health Sci Rep. 2021 Aug 13;4(3):e356. doi: 10.1002/hsr2.356 (PMC8362635; doi:10.1002/hsr2.356)
Supplement: Supplementary file 1 — Data S1. Supporting Information. [file HSR2-4-e356-s001.docx]

14,242 women aged 20 to 49 years

4,731 were selected for anthropometry

9,511 not selected for anthropometry

91 selected for anthropometry but not done

4,640 had their anthropometry done

1,356 were breastfeeding

3,284 were not breastfeeding

1,067 with normal BMI and underweight included in the bivariable and multivariable analyses

289 were obese and overweight

***Additional file Figure 1****: flow chat of sampling process*
